# COVID-19 self-testing in Nigeria: Stakeholders’ opinions and perspectives on its value for case detection

**DOI:** 10.1371/journal.pone.0282570

**Published:** 2023-04-13

**Authors:** Veronica A. Undelikwo, Sonjelle Shilton, Morenike Oluwatoyin Folayan, Oluwatoyin Alaba, Elena Ivanova Reipold, Guillermo Z. Martínez-Pérez

**Affiliations:** 1 Department of Sociology, University of Calabar, Calabar, Cross River State, Nigeria; 2 FIND, the Global Alliance for Diagnostics, Geneva, Switzerland; 3 Department of Child Dental Health, Obafemi Awolowo University, Ile-Ife, Osun State, Nigeria; 4 Institute of Public Health, Obafemi Awolowo University, Ile-Ife, Osun State, Nigeria; University of Foggia: Universita degli Studi di Foggia, ITALY

## Abstract

**Background:**

COVID-19 testing coverage is limited in Nigeria. Access to rapid SARS-CoV-2 antigen-detection self-testing kits may help improve the detection of asymptomatic and mildly symptomatic cases and increase the country’s low rate of SARS-CoV-2 testing. Before implementing self-testing in Nigeria, assessing the population’s perceptions regarding this approach is imperative. In mid-2021, an exploratory cross-sectional qualitative research was conducted to investigate stakeholders’ values and preferences for SARS-CoV-2 self-testing in Nigeria.

**Methods:**

In-person and online semi-structured interviews and focus group discussions with healthcare workers, representatives of civil society, and potential implementors of self-testing delivery programs were used to explore values and perceptions around access to conventional provider-initiated COVID-19 testing. Topics included the public’s values in relation to SARS-CoV-2 self-testing, the safe and effective use of SARS-CoV-2 self-testing, and likely actions upon receiving a positive SARS-CoV-2 self-test result. A thematic analysis approach was applied.

**Results:**

The 58 informants (29 female) reported that Nigeria has limited availability of conventional provider-delivered SARS-CoV-2 testing. While just a few informants were familiar with SARS-CoV-2 self-testing, they generally supported using self-testing as an approach that they felt could assist with early case detection and improve access to testing. Concerns relating to the use of self-testing mainly related to the ability of low-literate individuals to use and interpret the self-tests, the affordability of self-tests, equity of access, and the availability of healthcare system support for those who self-test positive.

**Conclusion:**

Although the Nigerian public perceive multiple benefits associated with access to SARS-CoV-2 self-testing, the perceived inefficiency of the national health service delivery system may limit the access of users of the kits to psychosocial and clinical support. Nevertheless, in Nigeria, where COVID-19 vaccine coverage is low and the risk of further waves of COVID-19 is high, self-testing may assist in the prompt detection of cases and contribute to halting the spread of the virus.

## Introduction

Coronavirus disease 2019 (COVID-19) is a novel airborne respiratory infection that has caused a global pandemic, resulting in more than 332 million infections and 5.5 million deaths to January 2022 [1]. Although vaccines against severe acute respiratory syndrome coronavirus 2 (SARS-CoV-2), the virus that causes COVID-19, can reduce the severity of infection, they do not eliminate the risk of infection [2]. There is a need for sustainable containment strategies to halt its transmission, especially in low- and middle-income countries (LMICs), where COVID-19 vaccine coverage remains low [3].

One effective strategy to help contain COVID-19 is community-wide testing to detect cases promptly. The most accurate technology for detecting SARS-CoV-2 is real-time reverse transcription polymerase chain reaction (RT-PCR) [4]. However, most LMICs have insufficient RT-PCR-equipped laboratories [5]. To facilitate community-level case identification, rapid SARS-CoV-2 antigen-detection tests (RADTs) represent an easy-to-perform solution for LMICs, although they are less sensitive than RT-PCR.

Even if availability of RT-PCR and RADTs improves, many asymptomatic cases of COVID-19 may go undetected [6]. To reduce this risk, self-tests for frequent home-use can enable people to detect SARS-CoV-2 infection without the direct assistance of healthcare professionals [7, 8]. While not yet widely introduced in most LMICs, SARS-CoV-2 self-testing devices have already been approved in some of the most populous nations such as China [8], the United States [9] and India [10]. In some LMICs, rapid self-tests for human immunodeficiency virus (HIV), malaria, and syphilis are already used [11–15]. The World Health Organization (WHO) has recently released recommendations for hepatitis C virus self-testing [16].

The acceptability of self-testing among the general population is usually high, as this approach can help to ensure higher levels of confidentiality and is usually more affordable than travelling to a clinic to receive a provider-delivered test [13, 17]. As with other self-testing devices, SARS-CoV-2 self-testing devices may be a feasible solution for governments with financial constraints to conduct mass screening for COVID-19, provided there are clear pathways to ensure that self-test users can adhere to appropriate behaviours post-self-testing, such as isolating at home and reporting a positive result.

In Nigeria, the country in West Africa that has been worst affected by the COVID-19 pandemic [18], the concept of individuals having access to self-testing for infectious diseases is not new. There is high acceptability of self-testing for HIV [19–21] and for malaria [22, 23]. As of 2022, SARS-CoV-2 self-testing devices were not yet regulated for public sale or distribution by health authorities in the country. In this context, access to self-testing may help increase the prompt detection of asymptomatic and mildly symptomatic COVID-19 cases and improve the country’s low rate of COVID-19 testing [24]. It could also reduce individuals’ resistance to seeking care as a result of the stigma associated with COVID-19 [25].

To issue recommendations for the issuing of regulatory guidance, public health practice, and marketing options around SARS-CoV-2 self-testing in Nigeria, it is imperative to understand the population’s culturally grounded perceptions regarding this approach as a complement to the professional use of RT-PCR and RADTs. In addressing this knowledge need, we conducted a qualitative inquiry to investigate the Nigerian public’s values and preferences around SARS-CoV-2 self-testing.

## Methods

### Study design and site

For this exploratory, cross-sectional qualitative inquiry, semi-structured interviews (SSIs) and focus group discussions (FGDs) were used as data collection techniques. The study was conducted by the Institute of Public Health, Obafemi Awolowo University, Ile-Ife with the support of FIND, the global alliance for diagnostics. This was an ancillary study to a larger population-based survey conducted in Nigeria between July and September 2021, which assessed the general public’s values and acceptance around SARS-CoV-2 self-testing (hereafter referred to as “self-testing”) [26].

### Population and sampling

The study population comprised three groups of decision-makers. First, healthcare workers (HCWs) were targeted because of their capacity to recommend self-testing to their patients and communities in their catchment areas. Spokespersons or representatives of various civil society organizations (RCSs) were targeted because of their capacity to influence community decision-making with regards to the utility of self-testing and guide people on what to do following a reactive self-test result. Potential implementers of self-testing delivery programs (PIs) were targeted because of their capacity to decide to pool financial and human resources to distribute self-testing at scale, for example in the workplaces they managed or in the geographies where they had jurisdiction to operate. Common inclusion criteria for all populations were: aged 18 years or older; willing to provide informed consent; fluent in English or Yoruba.

Efforts were made to ensure a diverse sample with regards to the informants’ gender, workplaces, socio-professional profiling, and areas of influence (i.e., based in a rural or urban setting, in the public or private sector). A purposive sampling approach was used to ensure the diversity of voices expected. Sex-disaggregated lists of at least 50 profiles per study population were produced. The study team, based in Obafemi Awolowo University (Ile-Ife, Osun State), used a variety of means (i.e., Google search engine, university reports, locally-available printed media, and websites of local authorities and other socio-professional councils or non-profit organizations) to source the names and contact details of all the persons whose profiles were proposed for recruitment. Subsequently, these sex-disaggregated lists were randomly rearranged using RANDOM.Org^®^. The study team in Ile-Ife contacted potential informants by phone, starting with the first name on each list. The persons who were reached by the study team were provided with information about the study aim, and those who expressed an interest were asked to participate in either an SSI or an FGD (but not in both).

### Data collection and processing

All informants gave written informed consent. Depending on their preferences, data collection was conducted via Zoom^®^ software or in-person at a place convenient for the informant and the interviewer. Each informant chose the language in which data collection was to be conducted.

The data collection was led by a mixed-gender team of experienced qualitative research assistants based in Ile-Ife. A 45-item guide, which was piloted in the Obafemi Awolowo University premises, was used for the SSIs and FGDs. The guide included questions and probes around six main topics: knowledge of conventional provider-delivered testing; values around self-testing; the public’s preferences for the delivery of self-testing; safe and effective use of self-testing; likely actions taken upon receiving a reactive or a non-reactive self-test result; and future prospects for the distribution of self-tests among the general public [26].

All encounters were audio-recorded. The recordings were transcribed verbatim into MS-Word^®^ files. Responses in Yoruba were translated into English. All transcripts were cross-checked by the analysts against the recordings for accuracy and completeness.

### Data analysis

Transcripts were uploaded into Quirkos^®^ software, and a thematic coding and analysis approach, guided by qualitative research methodologists Kielmann and colleagues’ recommendations [27], was applied. First, all transcripts were deductively coded using a pre-defined coding scheme [26]. Then, new codes were inductively created whenever an emerging theme was identified. In parallel with the coding, the analysts prepared personal memos and practiced reflexivity.

Iteratively with the coding, the dataset was analysed using a four-stage approach: transcript by transcript at first; followed by a theme-by-theme, sex-sensitive comparison of coded narratives across all transcripts and then by a theme-by-theme rural versus urban-sensitive comparison of coded narratives across all transcripts; and finishing with a comparison of key findings across the three study groups.

This article was prepared taking into consideration general insights and insights from isolated or deviant cases. The informants’ own words were used to prepare reports of the findings. Attention was paid to the analysts’ reflexive journals to ensure that no analysts’ informant biases were being introduced.

### Ethics statement

This study protocol and the informed consent documents were approved by the Health Research Ethics Committee of the Obafemi Awolowo University in Ile-Ife (Ref. IPH/OAU/12/1730). Before any data were collected, the informed consent documents were shared by email with the respective informants to give them more time to decide about their participation. All informants signed two copies of the information sheet and consent form, and they received a signed copy. Signed documents were collected by the SSI interviewers and FGD moderators. All signed documents were kept in a locked cabinet at the principal investigator’s office at Obafemi Awolowo University. Informants who attended the in-person FGDs were compensated for their transportation costs.

COREQ guidelines were considered in research reporting and dissemination. Additional information regarding the ethical, cultural, and scientific considerations specific to inclusivity in global research is included in the (S1 Checklist)

## Results

### Participants’ characteristics

Two FGDs and ten SSIs were conducted with each of the three study populations. On average, SSIs and FGDs lasted for 55 minutes and 122 minutes, respectively. In total, 58 decision-takers (29 female) participated. Their mean age was 45 years. Half of the informants were living or working in rural Osun State. Most informants (n = 55) had completed tertiary education (diploma, bachelors, or masters). Among the 19 HCWs, 5 were nurses. There was diversity in terms of the institutional representation of PIs and RCSs. To protect the anonymity of RCSs and PIs, the socio-demographic information presented in S1 Table only indicates their socio-professional sector of influence.

The findings are presented below as per the key themes identified during the analysis stage. Unless otherwise specified, the voices reported below reflect common opinions expressed across all study populations.

### Current COVID-19 testing

Testing for COVID-19 was not considered to be in great demand among mildly symptomatic people. Walk-in visits by community members to testing sites were described as not numerous because communities were perceived to be unable to afford the travel-related costs, to be “poorly educated” about COVID-19 symptoms, or to lack “motivation” to request testing. Other deterrents to testing identified included frequent delays in receiving test results, a generalised perception that COVID-19 is a low-risk disease, and fear of “isolation” and “stigmatisation”:

*It has to do with the early stigmatisation*. *Once someone is tested positive to COVID-19*, *the society and even the immediate family discriminates against him*, *and this has been a contributory factor for discouraging people to go and test*. (SSI 26, rural male PI)

Among other reasons given for the low demand for testing was the suggestion that “disbelief” about the existence of COVID-19 was commonplace. Misconceptions about COVID-19 being synonymous with malaria were also described:

*Many people still believe that COVID does not exist*, *that is just like malaria and that they don’t have to go for testing because if they are being diagnosed of COVID*: *that maybe is a death sentence*, *that they have to isolate them*. (SSI 19, urban female HCW)

All informants who had direct (e.g., collection of nasal or blood samples) or indirect (e.g., being a member of the state’s COVID-19 committee) experience of COVID-19 testing resided or worked in urban areas. While all HCWs were aware that COVID-19 could be diagnosed using RT-PCR and RADT, the majority of RCSs and PIs could not explain what diagnostic technologies for COVID-19 were available in their contexts.

Just a few urban HCWs expressed that they were directly involved with testing themselves. The majority of HCWs explained that the scarcity of COVID-19 diagnostic centres–together with facility staff being too busy caring for patients (of any condition)–limit the healthcare system’s capacity for community-based case detection. The shortage of trained professionals to conduct COVID-19 testing, poor availability of COVID-19 diagnostics, and limited access to personal protective equipment were other limiting factors for the routine testing of patients and their contacts. The RCSs also added language barriers, lack of privacy, and low monthly wages as barriers for the conduct of community-based testing by the healthcare system.

### Values associated to SARS-CoV-2 self-testing

Just three HCWs were aware of SARS-CoV-2 self-testing. These HCWs had learned about self-testing through social media, international scientific journals, and satellite television such as “CNN or Al Jazeera”.

Despite the general lack of awareness around self-testing, most informants considered that this approach could offer potential advantages. Self-testing was valued as an approach that would help the public to reduce costs, time, and other resources necessary to access facilities equipped with COVID-19 diagnostics. Self-testing was defined as a potentially private, convenient, and easy way to obtain a prompt diagnosis of COVID-19, to facilitate access to early treatment and, as a consequence, to reduce COVID-19-attributable mortality. All study groups suggested that self-testers who fear stigma would consider valuable the fact that they could keep their results confidential:

*One advantage is it will make the detection of the disease very easy because it could actually serve as a facilitator*, *because people will prefer to do the test themselves in the comfort of their homes instead of going out to health facility and then everybody starts looking at them and thinking that*: *“Does this person have COVID-19 or not*?*”* (SSI 5, rural female RCS)

Despite the identified positive values of self-testing, some potential disadvantages were also identified. Most importantly, as per the informants’ narratives, some potential end-users, especially low-literate individuals, may have reduced capacity to comply with the kits’ instructions and may be unable to interpret the results correctly. However, as one PI elaborated, if the kits are designed with full consideration of the country’s low literacy levels, most end-users will be able to adequately perform the self-test, in the same way that low-literate individuals with diabetes are able to use their glucose monitoring devices:

*Glucostix* [i.e., glucose strips by Bayer Diagnostics] *is there*, *and it is graded in different colour codes*. *That is the sort of thing to be done*, *so that even an illiterate*, *someone who is not educated*, *know that the moment you see red*, *it means danger*. *So*, *you don’t need to put figures there* [in the self-test kit’s user instructions]. *You can use colour codes*. (SSI 22, urban female PI)

### Preferences: Instructions, cost, specimen, distribution points

Information suggested for inclusion in the self-testing kit’s instructions included how to unpack, use and dispose of the kit; how to interpret the result; what the time interval before a repeat test should be; and what to do if the result is positive. To ensure ease of use, context-tailored step-by-step user instructions should be provided in English, Igbo, Hausa and Yoruba. Some RCSs and PIs noted that user instructions should also be provided in Braille.

To tackle the likely barrier of unaffordability, a few PIs and all RCSs opined that self-testing kits should be delivered free-of-charge. Conversely, some HCWs, PIs and RCSs opposed the distribution of free kits on the premise that the public, as one PI put it, “do not value what is free”. If the devices had to have a market price, the preferred maximum cost expressed by RCSs and PIs was Naira (N) 250 and N500, respectively (N100 is approximately US$0.25). HCWs held the most varied views with regards to pricing, with some suggesting prices ranging between N100 and N500 and others suggesting prices ranging between N1000 and N2500.

With regards to specimen type, the public’s preferred test specimens could be sputum, urine and saliva. Blood collection was considered “too invasive”, as it would require a trained professional to perform it, and could thus be, in the informants’ opinion, the least preferred specimen for self-testing:

*People are beginning to clamour for non-invasive procedures*. *I would love a situation whereby the use of saliva can be explored*. *Everybody spits all over the place*, *so we shouldn’t… Now what we are doing is a throat swab and everything*, *but if you have done that testing… you would know that “oh my God*!*” Especially the nasal one*, *it’s painful*. (SSI 22, female urban PI)

Regarding availability, it was suggested that a range of stakeholders from the public (e.g., healthcare workers), private not-for-profit (e.g., non-governmental organisations, NGOs), civil society organisation (CSO), and private for-profit (e.g., pharmacies, patent medicine vendors) sectors could be engaged with the distribution of self-tests. As per the informants’ suggestions, kits could be made available in churches, mosques, football fields, cinemas, and barbers’ salons, or through NGO/CSO community and house-to-house outreach programmes.

### Willingness to recommend self-testing

The majority of informants stated that they would recommend self-testing to the public, in their institutions and areas of influence, as they considered that this approach could lead to early commencement of treatment for COVID-19 for those who might need it. Nevertheless, the possibility of obtaining invalid results due to poor compliance with the test instructions was frequently mentioned by many informants as a factor that may discourage them from recommending self-testing.

Overall, the informants’ likelihood of recommending self-testing might also be influenced by the devices’ market price, availability in the communities, and accuracy. Some of the HCWs partaking in the FGDs claimed that their likelihood to recommend self-testing would be conditional on the kits indicating that they are approved by the National Agency for Food and Drug Administration and Control (NAFDAC, see: https://www.nafdac.gov.ng).

### Potential target users

Although there was consensus that the availability of self-testing may improve public interest in COVID-19 testing, it was also suggested that it would be mainly travellers who would prefer self-testing to avoid the “stress of doing a PCR”, that the “elites” would be among the first to use self-testing as they have more financial resources than the average Nigerian, and that urban dwellers would show more interest in self-testing than rural inhabitants. The HCWs added that some healthcare professionals would benefit from the regular use of self-testing if they are exposed to COVID-19 in the workplace.

Some RCSs and HCWs opined that there were no circumstances under which access to self-testing should be restricted. However, some PIs thought that “minors” should have limited access to self-testing. An urban, female PI thought that access to self-testing should be limited when there is “no longer an upsurge in infection rates” and the perception of risk associated with COVID-19 is low. Some HCWs added that to avoid misinterpretation of results or use of expired kits, elderly persons living alone and low-literate individuals should have limited access to self-testing.

### Risk of psychosocial harm

All study groups indicated that an indicator of the success of self-testing approaches for COVID-19 case identification would be self-testers’ communication of their positive results to health authorities. However, all groups also expressed that concerns of death and severe disease, forced isolation, and community-enacted stigma against the diseased were reasons for potential under-reporting. Some HCWs also noted that people’s concerns about health facility-induced stress, resulting from being passed through multiple departments to receive COVID-19 confirmatory testing and care, could also be a driver of under-reporting.

It was noted that “forced isolation” for those who receive a positive result might be feasible only for the “elites”. Most Nigerians may perceive home isolation as something to be “dreaded”, especially by those of “low socio-economic status who live in crowded spaces”. For many families, it could be impossible to stay at home until the end of their infection unless they receive support from an NGO/CSO in the form of food products or basic hygiene items or, as some PIs indicated, direct financial support. Despite isolation being a measure recommended by health authorities, the HCWs expressed empathy with individuals who might not be able to comply with this measure.

*They may wish to isolate but circumstances may not allow them*. *Like*, *if they are sharing rooms with members of their family*, *if they are not living in personal environment*, *they may not be able to isolate*. *So*, *the only thing they can do is for them to just protect themselves*, *or use their face mask*, *and they should ensure the people around them use their face mask*. (FGD 4 with urban HCWs)

Individuals’ non-compliance with isolation following a positive self-test result was not the biggest concern for many informants. Some HCWs thought that, although some people may use self-testing and refuse to disclose a positive result, there was still a likelihood that they would take precautions not to infect others. As one RCS noted, if he used a self-test device and its result were positive, he would communicate at work that he was “ill” and would protect his family members, but he would not report his result to his health facility.

The informants considered that those individuals who perceived that a positive result “means death” may be at risk of psychosocial ill health, while “resilient” individuals may be more likely to react in ways that protect others. Among the former, the impact might manifest as avoiding people, becoming “depressed”, suffering from insomnia, losing the ability to concentrate, or feeling “lonely” and “afraid of the unknown”.

*The person is going to test himself or herself*, *and then of course [is going to] know the result alone*, *which gives some confidentiality*. *However*, *the disadvantage is that it can lead to some mental issues*, *like depression and possibly suicidal tendency if not properly managed*. (SSI 23 with urban male PI)

The impact of a positive result on individuals might depend on their “personality”, level of education and location of residence. To some informants, the “common man” does not perceive COVID-19 to be “fatalistic”. The “breadwinners” in a household and people with comorbidities might be particularly concerned about receiving a positive result. It was also frequently expressed that women would react more positively to a positive result than men and young people who, as per some informants’ opinions, have generally poorer health-seeking behaviours than women.

There was consensus that a supportive environment may mitigate the risk of psychosocial harm following a positive self-test result. If end-users received pre- and post-test counselling, they would be “psychologically prepared” for a positive result. A few HCWs suggested that end-users be counselled in the use of the self-test kit and on how to link to COVID-19 care before receiving the kit. All groups stressed the need for sustained public education via outreach activities through churches, NGOs/CSOs, mosques, social media, or through television and radio broadcasts.

### Future prospects for the delivery of self-testing

All study groups expressed that treatment provision and contact-tracing following an end-user self-reporting in a clinic might be difficult due to a lack of adequate human and logistical resources. The Nigerian government should, in some PIs’ and HCWs’ opinions, strengthen the health sector to prevent the public from becoming disappointed with self-testing. This could be achieved by increasing the number of staff in healthcare facilities to cater to the number of end-users who might wish to manage a COVID-19 disease–irrespective of their symptoms–following a positive self-test result. Other steps should include improving health facilities’ existing staff capacity to manage cases effectively, irrespective of their severity; providing personal protective equipment to all staff tasked with direct management of COVID-19 cases; and increasing the number of facilities where end-users could both report a positive self-test result and receive confirmatory testing, post-test counselling and clinical care. PIs and RCSs identified the need for closer collaboration between health facility personnel and the community, including having “community development workers” to ensure that self-test users receive an appropriate response. Some PIs also noted that the government could promote self-testing uptake by providing concurrent opportunities for COVID-19 vaccination at self-testing distribution points, with simultaneous national policies mandating regular self-testing in work environments.

Other barriers to be addressed before the distribution of self-tests included the anticipated poor distribution of and unequal accessibility to self-testing throughout the country; poor awareness of the availability of self-testing; the likelihood of “hoarding” or stock-outs of self-testing; or, as one informant suggested, other security risks such as “kidnapping (of people distributing the kits)”.

### Public education on self-testing

It was proposed that the kits should be made affordable through government subsidy; be accessible from medical supply outlets in all communities; and be introduced to the public following adequate education, which could be sustained using community-adapted printed and web -based social media.

The “fear of death”, as expressed by a few RCSs, should not be used in promotional messages. Rather, public messaging should emphasise “responsibility to care”. Some RCSs suggested that public acceptability of self-testing may improve if, during public education, the government does not “insinuate” that their efforts in promoting self-testing are for “ulterior motives” (i.e., in reference to possible suspicions that government officials may be profiting from the introduction of self-testing in their communities).

## Discussion

This study harnessed the opinions of critical stakeholders who could be involved in the rollout of rapid SARS-CoV-2 antigen-detecting self-testing in Nigeria. These stakeholders included representatives of communities who might become the potential end-users of self-testing, healthcare workers who might advise patients and communities on self-testing usage, and implementers from the private and public sectors who might support the country’s continued access to self-testing and post-testing counselling and clinical care. Their voices can be considered proxies for the Nigerian public’s values and preferences towards self-testing as an approach to complement provider-delivered SARS-CoV-2 testing efforts.

We found that there was consensus across all stakeholders groups that self-testing would be of considerable value in helping to overcome some of the current individual-, health system-, and community-level barriers to accessing conventional provider-delivered testing. Nevertheless, the uptake and use of self-testing were not perceived by our informants to be free of challenges. Notably, our informants expressed concerns about the possibility of performance and interpretation errors and about the difficulties of self-isolating and of communicating the result following a reactive self-test. To overcome any potential risks associated with the misinterpretation of results, misuse of kits, or under-reporting of reactive results, the informants proposed strategies to promote the safe uptake of self-testing and to guarantee counselling and healthcare provision to those whose self-test result is interpreted as reactive. The strategies proposed are relevant for policy-making and implementation of self-testing delivery programmes in Nigeria as they can be helpful to plan distribution of self-testing to the public in an efficient way not only for patients, but also for the healthcare professionals supporting case detection at communities- and facilities-level.

Self-testing is an approach that offers opportunities for asymptomatic or mildly symptomatic individuals to rule out the possibility of having COVID-19. One of the values of self-testing identified by our informants was that it might reduce the burden on overstretched healthcare facilities. During the first year of the COVID-19 pandemic, the Nigerian healthcare system was overburdened by financial, human resources, and testing supply shortages [28, 29]. While plans must be instituted to accommodate a likely increase in the number of self-testers that may visit their nearest clinic requesting confirmatory testing, these informants’ opinions about the potential of self-testing to alleviate the burden on the healthcare system were shared by decision-takers who participated in a similar self-testing acceptability study in Indonesia [30]. To address potential increases of asymptomatic self-test users’ requests of confirmatory testing, it may be recommended that users interpret a reactive self-test result as a positive diagnosis of SARS-CoV-2 infection. However, there is no consensus in this aspect amongst other resource-constrained countries. Whilst Nigeria lacks official recommendations on self-testing; the Government of India issued guidance advising self-test users to immediately isolate if they receive a self-test result [10]; and, health authorities in Brazil [31] and Indonesia [32] warn users that self-test kits are not diagnostic tools and they should request confirmatory testing in a health facility if they receive a reactive self-test result.

As expressed by our informants, some of the structural barriers to facility-based COVID-19 testing that were identified, such as the cost of healthcare, unavailability of diagnostics and therapies, and rejection of the “diseased” by certain sections of the public, might affect the uptake of self-testing. Policy- and programme-makers can, in considering these barriers, plan actions to mitigate their impact, such as: subsidize the cost of healthcare for the most deprived households, establish public-private partnerships to scale up availability of confirmatory diagnosis, and continue educating the public on the need to not stigmatise the individuals who acquire a SARS-CoV-2 infection and seek care. These barriers of access to facility-based COVID-19 testing are not specific to Nigeria, as they have also been reported in geographies as diverse as the United States [33], Ghana [34], or Jordan [35]. These structural barriers are among the barriers that also make access to HIV self-testing difficult for some vulnerable groups in resource-constrained countries [36]. In Nigeria, the cost of healthcare already hampers the uptake of and adherence to HIV services [37] and preventive care [38], and it is a critical consideration for the provision of laboratory services [39].

The informants of our study suggested that the cost of self-testing devices should be subsidised, although some expressed concern that the social and financial costs of isolation might be a greater worry than the cost of self-testing. For individuals where cost is a concern, their social environment and living conditions may make it virtually impossible to self-isolate and prevent further transmission of the virus. Policy and programme actions are therefore needed not only to ensure affordable access to self-testing kits but also to support individuals to self-isolate upon testing positive. Such support might include sustained social protection services such as cash transfers, food vouchers and subsidised utilities for families and individuals whose sources of income risk being disrupted due to self-isolation. The same types of support measures from authorities to individuals self-isolating after receiving a reactive self-test result were also reported in the self-testing acceptability study carried out in Indonesia [30].

In the absence of social safety nets, as some of our informants reflected, some individuals might find it difficult, irrespective of their intrinsic healthcare-seeking motivations, to adhere to the recommended behaviours that should follow a reactive self-test result. In this regard, Nigeria could consider its own experiences with the distribution of HIV self-testing [40, 41], and could consider how access to SARS-CoV-2 self-testing was fully subsidised in countries such as Austria [42] or Greece [43]. Evaluations of the cost-effectiveness and impact of these self-testing programmes might provide guidance for Nigerian health authorities to take evidence-based decisions regarding up to what extend self-test devices could be subsidised in the country, what type of safety nets provision could be prioritised to promote home isolation and contact warning after interpreting a self-test result as reactive, as well as to how to plan a socio-economic evaluation of the delivery of self-testing to the public as a complementary approach to curve down the SARS-CoV-2 incidence in the Nigerian territory.

Concerns about “hoarding” and stock-outs were expressed in our study. A recent review has identified that limited stocks of essential diagnostics for COVID-19 have been frequent in resource-constrained countries [44]. For any future implementation of self-testing delivery programs in Nigeria and similar health resource-constrained settings, it will be important to identify which distribution and accountability models will be the most cost-effective in making self-testing available (and affordable) in areas where the communities have concerns regarding the governance of health product supplies. As hinted at by some HCW informants, the Nigerian government (i.e., referring to NAFDAC and other public health authorities) could make a key contribution by passing stringent regulations on self-test distribution and quality assurance, to mitigate the risk of unavailability of quality self-test kits. Lessons learnt from countries such as Spain, where pharmacies struggled to distribute self-testing kits during the peak of the Omicron variant of concern wave by the end of 2021, might provide some guidance. In Spain, the government passed regulations to set a ceiling of 2.94€ in the price of self-tests so that low-income households could access them [45]. In addition to considering these experiences from high-income countries, Nigeria can also consider the lessons learnt from its myriad actions to ensure availability of quality antiretrovirals for its citizens living with HIV [46, 47]. In the West African region, Nigeria is indeed among the countries which have developed a robust pre-existing infrastructure and technical capacity to thoroughly plan and secure the availability of quality self-test kits to the public.

In determining the most cost-effective models for the distribution of self-tests, other, emotionally related factors interact with cost and the regulatory framework. The psycho-social burden of receiving a reactive self-test result must be considered. As with HIV infection, COVID-19 is associated with stigma [48], which implies that for distribution models to be cost-effective they must include provisions to mitigate the fear of being discriminated or stigmatised for having COVID-19 and, as a consequence, incurring social and economic loss. In the absence of provision of psychosocial support and clear pathways for linkage to post-self-test care, even the best distribution models may fail. Our study emphasises the need for pre- and post-self-test counselling provision, as well as for the engagement of various stakeholders from the public and private not-for-profit healthcare provision sectors to support provision outside of the regular healthcare system. Willingness to request counselling provision following a reactive self-test result was also suggested by participants in SARS-CoV-2 self-tests acceptability surveys in Indonesia [49], and in Kenya [50].

The stress associated with the possibility of “forced isolation”, as some informants termed this measure recommended by health authorities for those infected with SARS-CoV-2 and not requiring hospitalization, must be acknowledged as one of the most impactful impediments to testing. This is a concern for many cisgender men who are burdened with the need to provide care for their family as the sole breadwinner in many households, as well as for many cisgender women who work in the informal economy and rely on their daily wages to provide for their children [51]. The potential impact of isolation among sexual and gender minorities was not discussed in our study, however there is evidence from Indonesia [30] or the United States [52] that these minorities may face considerable socio-economic constraints to cope with isolation. The impact in children was also not discussed by our study informants. In a country such as Nigeria, with several regions severely affected by high rates of malnutrition among children under 5 years [53], a debate is urgently needed about which measures would be the most effective and acceptable, to both society and the health authorities, to ensure that people who are infected but who cannot isolate will be able to provide for their households without the risk of transmitting SARS-CoV-2 to others.

Our informants also suggested that “forced isolation” was a more significant concern than fear of morbidity. This study did not provide an understanding of why there might be a low perception of risk (i.e., individuals’ judgements about and evaluations of hazards to which they may be exposed) of COVID-19 disease among some Nigerians, although this low perception of risk is also a barrier to the use of self-testing. Nevertheless, what this study has identified is the need to tailor appropriate risk communication and education to enable individuals to understand their risks when resorting to traditional medicine in the absence of a confirmed malaria or a COVID-19 diagnosis, or when self-managing COVID-19 without having warned their close contacts.

Finally, gender norms are another structural factor that may affect the use of self-tests and which cannot be transformed in the short-term. Self-testing distribution models must include targeted strategies to encourage the uptake of self-testing by men and adolescents who, as per our respondents’ voices, are perceived to exhibit limited use of health services or to be individuals who have worse healthcare behaviours than women. Lessons on entry strategies for self-testing could be learned from the introduction of HIV self-testing that specifically targeted men [19].

### Strengths and limitations

Our study has some limitations. First, the informants were recruited from both urban and rural areas in Nigeria, and diversity regarding gender identities, location of work, and socio-professional profiles was ensured. However, this was a qualitative study, and the informants’ insights may not represent all possible opinions in the country. Nevertheless, our findings offered insights that might characterise the specific groups represented in our sample. Second, some data collection encounters were carried out in person and others via Zoom^®^. While the content of interviews conducted online and in-person was similar, the interviewers felt that it was easier to build rapport with the interviewees when partaking in face-to-face encounters. Third, the possibility that informants interviewed via Zoom^®^ changed their narratives due to privacy or confidentiality concerns cannot be disregarded.

## Conclusion

In conclusion, based on the stakeholders’ opinions which were considered in our qualitative inquiry, facilitating the use of SARS-CoV-2 self-testing to increase COVID-19 case detection in Nigeria will require multiple layers of planning, ranging from the active engagement of policymakers to develop regulations and strategies for the rollout of a national self-testing programme, to capacity-building of health institutions to manage the increased demand that may result from the rollout, to the active engagement of communities and community decision-making platforms to allay fears and to promote the effective use of self-testing. While the public may perceive that access to self-testing will be beneficial in the long-term, healthcare institutions must be prepared to provide appropriate psychosocial and clinical support to self-testers. For a populous country like Nigeria, where COVID-19 vaccine coverage remains low, self-testing holds promise for allowing communities themselves to promptly detect cases and contribute to halting the spread of the SARS-CoV-2 in the region. Self-testing will only be beneficial in Nigeria if strategies are deployed to motivate adherence to recommended behaviours, such as isolation, warning close contacts, and the use of face masks, by symptomatic and asymptomatic individuals upon receiving a reactive self-test result.

## Supporting information

S1 Checklist Inclusivity in global research. (DOCX)

S1 Table Participants’ sociodemographic characteristics. (DOCX)

## References

[1] World Health Organization. Coronavirus disease (COVID-19) pandemic Geneva: World Health Organization; 2021 [Available from: https://www.who.int/emergencies/diseases/novel-coronavirus-2019

[2] Science Brief: COVID-19 Vaccines and Vaccination 2021 [Available from: https://www.cdc.gov/coronavirus/2019-ncov/science/science-briefs/fully-vaccinated-people.html

[3] World Health Organization. WHO Director-General’s opening remarks at the media briefing on COVID-19–11 March 2020 Geneva: World Health Organization; 2020 [Available from: https://www.who.int/director-general/speeches/detail/who-director-general-s-opening-remarks-at-the-150th-session-of-the-executive-board-24-january-2022

[4] Yüce M, Filiztekin E, Özkaya KG. COVID-19 diagnosis -A review of current methods. Biosens Bioelectron. 2021;172:112752. doi: 10.1016/j.bios.2020.112752 33126180 PMC7584564

[5] Giri AK, Rana DR. Charting the challenges behind the testing of COVID-19 in developing countries: Nepal as a case study. Biosafety and Health. 2020;2(2):53–6. 10.1016/j.bsheal.2020.05.002 PMC7219426 38620322

[6] Oran DP, Topol EJ. Prevalence of Asymptomatic SARS-CoV-2 Infection: A Narrative Review. Ann Intern Med. 2020;173(5):362–7. doi: 10.7326/M20-3012 32491919 PMC7281624

[7] Hengel B, Causer L, Matthews S, Smith K, Andrewartha K, Badman S, et al. A decentralised point-of-care testing model to address inequities in the COVID-19 response. Lancet Infect Dis. 2021;21(7):e183–e90. doi: 10.1016/S1473-3099(20)30859-8 33357517 PMC7758179

[8] National Medical Products Administration. China approves antigen tests, 14th March 2022. http://english.nmpa.gov.cn/2022-03/14/c_725341.htm Published March 2022. Accessed October 11, 2022.

[9] U.S. Food and Drug Administration. Coronavirus (COVID-19) Update: FDA Authorizes Antigen Test as First Over-the-Counter Fully At-Home Diagnostic Test for COVID-19 2020 [Available from: https://www.fda.gov/news-events/press-announcements/coronavirus-covid-19-update-fda-authorizes-antigen-test-first-over-counter-fully-home-diagnostic

[10] Indian Council of Medical Research. COVID-19 Home Testing using Rapid Antigen Tests (RATs) Indian Council of Medical Research; 2021 [Available from: https://www.icmr.gov.in/pdf/covid/kits/archive/COVID_Home_Test_Kit_08112021.pdf

[11] Ibitoye M, Frasca T, Giguere R, Carballo-Diéguez A. Home testing past, present and future: lessons learned and implications for HIV home tests. AIDS Behav. 2014;18(5):933–49. doi: 10.1007/s10461-013-0668-9 24281697 PMC3988264

[12] Kabaghe AN, Visser BJ, Spijker R, Phiri KS, Grobusch MP, van Vugt M. Health workers’ compliance to rapid diagnostic tests (RDTs) to guide malaria treatment: a systematic review and meta-analysis. Malar J. 2016;15:163. doi: 10.1186/s12936-016-1218-5 26979286 PMC4791859

[13] Sy TRL, Padmawati RS, Baja ES, Ahmad RA. Acceptability and feasibility of delegating HIV counseling and testing for TB patients to community health workers in the Philippines: a mixed methods study. BMC Public Health. 2019;19(1):185. doi: 10.1186/s12889-019-6497-7 30760257 PMC6375216

[14] Kpokiri EE, Marley G, Tang W, Fongwen N, Wu D, Berendes S, et al. Diagnostic Infectious Diseases Testing Outside Clinics: A Global Systematic Review and Meta-analysis. Open Forum Infect Dis. 2020;7(10):ofaa360. doi: 10.1093/ofid/ofaa360 33072806 PMC7545117

[15] Wang C, Cheng W, Li C, Tang W, Ong JJ, Smith MK, et al. Syphilis Self-testing: A Nationwide Pragmatic Study Among Men Who Have Sex With Men in China. Clin Infect Dis. 2020;70(10):2178–86. doi: 10.1093/cid/ciz603 31260513 PMC7201417

[16] World Health Organization. Recommendations and guidance on hepatitis C virus self-testing: web annex D: values and preferences on hepatitis C virus self-testing. Geneva: World Health Organization; 2021 2021.

[17] Nguyen LT, Nguyen VTT, Le Ai KA, Truong MB, Tran TTM, Jamil MS, et al. Acceptability and Usability of HCV Self-Testing in High Risk Populations in Vietnam. Diagnostics (Basel). 2021;11(2). doi: 10.3390/diagnostics11020377 33672241 PMC7926709

[18] Osayomi T, Adeleke R, Taiwo OJ, Gbadegesin AS, Fatayo OC, Akpoterai LE, et al. Cross-national variations in COVID-19 outbreak in West Africa: Where does Nigeria stand in the pandemic? Spatial Information Research. 2020:1–9.

[19] Hamilton A, Thompson N, Choko AT, Hlongwa M, Jolly P, Korte JE, et al. HIV Self-Testing Uptake and Intervention Strategies Among Men in Sub-Saharan Africa: A Systematic Review. Front Public Health. 2021;9:594298. doi: 10.3389/fpubh.2021.594298 33681120 PMC7933016

[20] Iliyasu Z, Kassim RB, Iliyasu BZ, Amole TG, Nass NS, Marryshow SE, et al. Acceptability and correlates of HIV self-testing among university students in northern Nigeria. Int J STD AIDS. 2020;31(9):820–31. doi: 10.1177/0956462420920136 32623978

[21] Brown B, Folayan MO, Imosili A, Durueke F, Amuamuziam A. HIV self-testing in Nigeria: public opinions and perspectives. Glob Public Health. 2015;10(3):354–65. doi: 10.1080/17441692.2014.947303 25186234

[22] Population Council. Feasibility and acceptability of HIV self-testing among men who have sex with men in Nigeria 2018 [Available from: https://www.popcouncil.org/uploads/pdfs/2018HIV_SelfTestingMSMNigeria.pdf

[23] Jegede AS, Oshiname FO, Sanou AK, Nsungwa-Sabiiti J, Ajayi IO, Siribié M, et al. Assessing Acceptability of a Diagnostic and Malaria Treatment Package Delivered by Community Health Workers in Malaria-Endemic Settings of Burkina Faso, Nigeria, and Uganda. Clin Infect Dis. 2016;63(suppl 5):S306–s11. doi: 10.1093/cid/ciw630 27941109 PMC5146702

[24] Al-Mustapha AI, Tijani AA, Oyewo M, Ibrahim A, Elelu N, Ogundijo OA, et al. Nigeria’s race to zero COVID-19 cases: True disease burden or testing failure? J Glob Health. 2021;11:03094. doi: 10.7189/jogh.11.03094 34408855 PMC8364253

[25] World Health Organization. Social stigma threatens COVID-19 response but patients heal faster with everyone’s support Geneva: World Health Organization; 2020 [Available from: https://www.afro.who.int/news/social-stigma-threatens-covid-19-response-patients-heal-faster-everyones-support

[26] Shilton S, Ivanova Reipold E, Roca Álvarez A, Martínez-Pérez GZ. Assessing Values and Preferences Toward SARS-CoV-2 Self-testing Among the General Population and Their Representatives, Health Care Personnel, and Decision-Makers: Protocol for a Multicountry Mixed Methods Study. JMIR Res Protoc. 2021;10(11):e33088. doi: 10.2196/33088 34726608 PMC8629348

[27] Kielmann K, Cataldo F, Seeley J. Introduction to Qualitative Research Methodology: DFID; 2011.

[28] Aregbeshola B, Folayan MO. Nigeria’s financing of health care during the COVID-19 pandemic: Challenges and recommendations. World Med Health Policy. 2022;14(1):195–204. doi: 10.1002/wmh3.484 34909238 PMC8661625

[29] Odusanya OO. Nigeria in the COVID Era: Health System Strengthening for National Security and Prosperity. Niger Postgrad Med J. 2022;29:192–7. doi: 10.4103/npmj.npmj_106_22 35900454

[30] Thomas C, Shilton S, Thomas C, Mone Iye C, Martínez-Pérez GZ. COVID-19 self-testing, a way to “live side by side with the coronavirus”: results from a qualitative study in Indonesia. PLoS Global Public Health (In press). doi: 10.1371/journal.pgph.0000514 36962512 PMC10021662

[31] GOV.BR Governo Federal. Resolução–RDC N° 595, de 28 de Janeiro de 2022 2022 [Available from: https://www.in.gov.br/web/dou/-/resolucao-rdc-n-595-de-28-de-janeiro-de-2022-376825970.

[32] Perhimpunan Dokter Specialis Patologi Klinic Dan Kedokteran Laboratorium Indonesia (PDS PatKLIn). Self-testing Covid-19 Rapid Test Antigen: PDS PatKLIn, Jakarta; 30 March 2022.

[33] Kim SJ, Watson K, Khare N, Shastri S, Da Goia Pinto CL, Nazir NT. Addressing Racial/Ethnic Equity in Access to COVID-19 Testing Through Drive-Thru And Walk-In Testing Sites in Chicago. Med Res Arch. 2021 May;9(5):2430. doi: 10.18103/mra.v9i5.2430 Epub 2021 May 25. ; PMCID: PMC8186439. 34109272 PMC8186439

[34] Ha S, Yangchen S, Assan A. COVID-19 Testing: A Qualitative Study Exploring Enablers and Barriers in the Greater Accra Region, Ghana. Front Public Health. 2022 Jul 12;10:908410. doi: 10.3389/fpubh.2022.908410 ; PMCID: PMC9322666. 35903391 PMC9322666

[35] Shahrour G, Jaradat D, Dardas LA. Barriers related to COVID-19 testing intention. Public Health Nurs. 2021 Nov;38(6):978–983. doi: 10.1111/phn.12950 Epub 2021 Jul 27. ; PMCID: PMC8447434. 34313354 PMC8447434

[36] Rivera AS, Hernandez R, Mag-Usara R, Sy KN, Ulitin AR, O’Dwyer LC, et al. Implementation outcomes of HIV self-testing in low- and middle- income countries: A scoping review. PLoS One. 2021 May 3;16(5):e0250434. doi: 10.1371/journal.pone.0250434 33939722 PMC8092786

[37] Ahonkhai AA, Regan S, Idigbe I, Adeniyi O, Aliyu MH, Okonkwo P, et al. The impact of user fees on uptake of HIV services and adherence to HIV treatment: Findings from a large HIV program in Nigeria. PLoS One. 2020;15(10):e0238720. doi: 10.1371/journal.pone.0238720 33031440 PMC7544141

[38] Ofoli JNT, Ashau-Oladipo T, Hati SS, Ati L, Ede V. Preventive healthcare uptake in private hospitals in Nigeria: a cross-sectional survey (Nisa premier hospital). BMC Health Serv Res. 2020 Apr 1;20(1):273. doi: 10.1186/s12913-020-05117-5 32238153 PMC7114808

[39] Olutuase VO, Iwu-Jaja CJ, Akuoko CP, Adewuyi EO, Khanal V. Medicines and vaccines supply chains challenges in Nigeria: a scoping review. BMC Public Health. 2022 Jan 5;22(1):11. doi: 10.1186/s12889-021-12361-9 34986820 PMC8727467

[40] Iwelunmor J, Ezechi O, Obiezu-Umeh C, Gbaja-Biamila T, Musa AZ, Nwaozuru U, et al. Enhancing HIV Self-Testing Among Nigerian Youth: Feasibility and Preliminary Efficacy of the 4 Youth by Youth Study Using Crowdsourced Youth-Led Strategies. AIDS Patient Care STDS. 2022 Feb;36(2):64–72. doi: 10.1089/apc.2021.0202 35147463 PMC8861905

[41] Tun W, Vu L, Dirisu O, Sekoni A, Shoyemi E, Njab J, et al. Uptake of HIV self-testing and linkage to treatment among men who have sex with men (MSM) in Nigeria: A pilot programme using key opinion leaders to reach MSM. J Int AIDS Soc. 2018 Jul;21 Suppl 5(Suppl 5):e25124. doi: 10.1002/jia2.25124 ; PMCID: PMC6055125. 30033680 PMC6055125

[42] Willeit P, Bernar B, Zurl C, Al-Rawi M, Berghold A, Bernhard D, et al. Sensitivity and specificity of the antigen-based anterior nasal self-testing programme for detecting SARS-CoV-2 infection in schools, Austria, March 2021. Euro Surveill. 2021 Aug;26(34):2100797. doi: 10.2807/1560-7917.ES.2021.26.34.2100797 34448449 PMC8393891

[43] Government of Greece (GOV.GR). Οδηγίες προς τους Πολίτες (Instructions to citizens). [Available from: https://self-testing.gov.gr

[44] Maluleke K, Musekiwa A, Kgarosi K, Gregor EM, Dlangalala T, Nkambule S, et al. A Scoping Review of Supply Chain Management Systems for Point of Care Diagnostic Services: Optimising COVID-19 Testing Capacity in Resource-Limited Settings. Diagnostics (Basel). 2021 Dec 8;11(12):2299. doi: 10.3390/diagnostics11122299 34943536 PMC8700402

[45] Boletín Oficial del Estado (BOE). Resolución de 13 de enero de 2022. BOE 14 Jan 2022, Num. 12, sec. I, p. 3432. [Accesible in: https://www.boe.es/eli/es/res/2022/01/13/(1)/con

[46] Bautista-Arredondo S, Colchero MA, Amanze OO, La Hera-Fuentes G, Silverman-Retana O, Contreras-Loya D, et al. Explaining the heterogeneity in average costs per HIV/AIDS patient in Nigeria: The role of supply-side and service delivery characteristics. PLoS One. 2018 May 2;13(5):e0194305. doi: 10.1371/journal.pone.0194305 29718906 PMC5931468

[47] Kwaghe AV, Ilesanmi OS, Amede PO, Okediran JO, Utulu R, Balogun MS. Stigmatization, psychological and emotional trauma among frontline health care workers treated for COVID-19 in Lagos State, Nigeria: a qualitative study. BMC Health Serv Res. 2021;21(1):855. doi: 10.1186/s12913-021-06835-0 34419034 PMC8380097

[48] Thomas C, Shilton S, Thomas C, Batheja D, Goel S, Mone Iye C, et al. Values and preferences of the general population in Indonesia in relation to rapid COVID-19 antigen self-tests: A cross-sectional survey. Trop Med Int Health. 2022 May;27(5):522–536. doi: 10.1111/tmi.13748 Epub 2022 Apr 5. ; PMCID: PMC9115524. 35332616 PMC9115524

[49] Manguro G, Shilton S, Omenda S, Owira P, Batheja D, Banerji A, et al. Are Kenyans Likely to Use COVID-19 Self-Testing Kits? Results from a Cross-Sectional Survey. International Journal of Public Health. 2022 (iFirst). doi: 10.3389/ijph.2022.1604918 36090834 PMC9459853

[50] Olu-Owolabi FE, Amoo E, Samuel O, Oyeyemi A, Adejumo G. Female-dominated informal labour sector and family (in) stability: The interface between reproduction and production. Cogent Arts & Humanities. 2020;7(1):1788878.

[51] National Bureau of Statistics, UNICEF, Saving One Million Lives Programme for Results. Report–The Nutrition and Health Situation in Nigeria. June 2018: UNICEF; 2020 [Available from: https://www.unicef.org/nigeria/media/2181/file/Nigeria-NNHS-2018.pdf

[52] Krueger EA, Barrington-Trimis JL, Unger JB, Leventhal AM. Sexual and Gender Minority Young Adult Coping Disparities During the COVID-19 Pandemic. J Adolesc Health. 2021;69(5):746–753. doi: 10.1016/j.jadohealth.2021.07.021 34412952 PMC8456158

[53] Aduh U, Folayan MO, Afe A, Onyeaghala AA, Ajayi IO, Coker M, et al. Risk perception, public health interventions, and Covid-19 pandemic control in sub-saharan Africa. Journal of Public Health in Africa. 2020.

