# Supplementary material for: COVID-19 self-testing in Nigeria: Stakeholders’ opinions and perspectives on its value for case detection
Source: PLoS One. 2023 Apr 13;18(4):e0282570. doi: 10.1371/journal.pone.0282570 (PMC10101386; doi:10.1371/journal.pone.0282570)
Supplement: S1 Table — (DOCX) [file pone.0282570.s002.docx]

Supplementary Material

# Table 1: Participants’ characteristics

| **Population** | **Encounter** | **Location** | **Sex** | **Age range** | **Sector Profile** |
| --- | --- | --- | --- | --- | --- |
| Representatives of Civil Society groups (RCSs) | IDI 1 | Rural | Female | >55 | NGO (HIV/TB/COVID-19) |
|  | IDI 2 | Rural | Male | 36-55 | NGO (HIV/TB) |
|  | IDI 3 | Rural | Male | 36-55 | News Media |
|  | IDI 4 | Rural | Male | >55 | Trade Union |
|  | IDI 5 | Rural | Female | 36-55 | Entrepreneur (Poultry) |
|  | IDI 6 | Urban | Male | 36-55 | NGO (Health, general) |
|  | IDI 7 | Urban | Female | >55 | Religious leader (Christian) |
|  | IDI 8 | Urban | Female | 36-55 | NGO (Children, Women welfare) |
|  | IDI 9 | Urban | Male | 36-55 | NGO (Cancer) |
|  | IDI 10 | Urban | Female | >55 | Education (Primary School) |
|  | FGD 1 | Rural | Male | 18-35 | Industry (Vineyard) |
|  |  | Rural | Male | 36-55 | NGO (Health, general) |
|  |  | Rural | Female | >55 | NGO (HIV) |
|  |  | Rural | Female | 36-55 | Traditional (Mentor Mother) |
|  |  | Rural | Female | 36-55 | NGO (Children welfare) |
|  | FGD 2 | Urban | Male | 18-35 | NGO (TB) |
|  |  | Urban | Male | >55 | NGO (HIV) |
|  |  | Urban | Male | 36-55 | NGO (Youth welfare) |
|  |  | Urban | Female | >55 | Traditional (Women leader) |
|  |  | Urban | Female | 36-55 | Religious entity (Christian) |
| Health Care Workers (HCWs) | IDI 11 | Rural | Female | 36-55 | Public Health Worker |
|  | IDI 12 | Rural | Female | 36-55 | Community Health Officer |
|  | IDI 13 | Rural | Female | 36-55 | Community Health Officer |
|  | IDI 14 | Rural | Male | 36-55 | Laboratory Technician |
|  | IDI 15 | Rural | Male | 36-55 | Community Health Officer |
|  | IDI 16 | Urban | Female | 18-35 | Nurse |
|  | IDI 17 | Urban | Male | 36-55 | Physician |
|  | IDI 18 | Urban | Male | 18-35 | Nurse |
|  | IDI 19 | Urban | Female | 18-35 | Nurse |
|  | IDI 20 | Urban | Male | 36-55 | Nurse |
|  | FGD 3 | Rural | Female | 36-55 | Community Health Officer |
|  |  | Rural | Male | 36-55 | Health record Technician |
|  |  | Rural | Male | 36-55 | Community Health Officer |
|  |  | Rural | Male | 18-35 | Community Health Officer |
|  | FGD 4 | Urban | Female | 36-55 | Physician |
|  |  | Urban | Male | 18-35 | Physician |
|  |  | Urban | Female | 18-35 | Community Health Officer |
|  |  | Urban | Male | 36-55 | Medical Laboratory Scientist |
|  |  | Urban | Female | 36-55 | Nurse |
| Potential COVID-19 self-testing implementers (PIs) | IDI 21 | Urban | Female | 36-55 | NGO (Women’s health) |
|  | IDI 22 | Urban | Female | >55 | Research (Bioethics Committee) |
|  | IDI 23 | Urban | Male | 36-55 | Professional Society (Public Health) |
|  | IDI 24 | Urban | Female | 36-55 | Health Ministry (Administration) |
|  | IDI 25 | Urban | Female | 36-55 | Education (Nursing) |
|  | IDI 26 | Rural | Male | 36-55 | NGO (Disabilities) |
|  | IDI 27 | Rural | Male | 18-35 | NGO (HIV) |
|  | IDI 28 | Rural | Male | >55 | Religious leader (Christian) |
|  | IDI 29 | Rural | Female | 18-35 | NGO (HIV) |
|  | IDI 30 | Rural | Female | 36-55 | NGO (Health, general) |
|  | FGD 5 | Rural | Male | >55 | Landlords Community |
|  |  | Rural | Male | 36-55 | NGO (Disabilities) |
|  |  | Rural | Female | 18-35 | NGO (Disabilities) |
|  |  | Rural | Female | 18-35 | NGO (Health, general) |
|  |  | Rural | Female | 18-35 | NGO (Youth development) |
|  | FGD 6 | Urban | Male | >55 | Industry (Health products, patents) |
|  |  | Urban | Female | 36-55 | NGO (Women’s health) |
|  |  | Urban | Male | 36-55 | Education (Medicine) |
|  |  | Urban | Male | 18-35 | NGO (Youth development) |
| Acronyms:  NGO: Non-governmental organization/Civil society-based organization; HIV: Human Immunodeficiency Virus; TB: Tuberculosis | | | | | |
